# The Role of JAK/STAT Pathway in Fibrotic Diseases: Molecular and Cellular Mechanisms

**DOI:** 10.3390/biom13010119

**Published:** 2023-01-06

**Authors:** Jia Liu, Faping Wang, Fengming Luo

**Affiliations:** 1 Department of Pulmonary and Critical Care Medicine, West China Hospital, Sichuan University, Chengdu 610041, China; 2 Laboratory of Pulmonary Immunology and Inflammation, Frontiers Science Center for Disease-related Molecular Network, West China Hospital, Sichuan University, Chengdu 610041, China

**Keywords:** Janus kinases (JAK), signal transducer and activator of transcription (STAT), cytokines, fibrosis, fibroblast, inhibitor

## Abstract

There are four members of the JAK family and seven of the STAT family in mammals. The JAK/STAT molecular pathway could be activated by broad hormones, cytokines, growth factors, and more. The JAK/STAT signaling pathway extensively mediates various biological processes such as cell proliferation, differentiation, migration, apoptosis, and immune regulation. JAK/STAT activation is closely related to growth and development, homeostasis, various solid tumors, inflammatory illness, and autoimmune diseases. Recently, with the deepening understanding of the JAK/STAT pathway, the relationship between JAK/STAT and the pathophysiology of fibrotic diseases was noticed, including the liver, renal, heart, bone marrow, and lung. JAK inhibitor has been approved for myelofibrosis, and subsequently, JAK/STAT may serve as a promising target for fibrosis in other organs. Therefore, this article reviews the roles and mechanisms of the JAK/STAT signaling pathway in fibrotic diseases.

## 1. Introduction

Fibrosis is an uncontrolled tissue repair process after injury or inflammation, eventually causing organ failure [1]. The fibrotic lesion occurs on multiple organs, including the bone marrow, liver, kidney, heart, lung, and skin. Chronic inflammatory diseases are vulnerable to fibrosis [2,3,4]. Fibrosis is usually accompanied by an overreactive inflammatory response and enriched cytokines [5]. However, limited therapies are available for patients with fibrosis at present. Twelve years ago, Janus kinases (JAK) inhibitors were gradually approved for bone marrow fibrosis and other inflammatory diseases, such as rheumatoid arthritis, ulcerative colitis, and psoriatic arthritis, etc. The JAK/signal transducer and activator of transcription (STAT) signaling pathway is a cascade of responses critical for the signal transduction of multiple cytokines and growth factors in vivo [6,7]. This pathway regulates gene expression as well as cell activation, proliferation, differentiation, apoptosis, autophagy, and endoplasmic reticulum stress [8,9]. The proline-rich domain of the cytokine receptor proximal to the membrane is associated with the composition of JAK [10]. The binding of the cytokine receptor to the ligand induces a conformational change in the receptor, resulting in the entry of the JAK molecule into the proximal binding site of the receptor. JAK can then cause the phosphorylation of tyrosine residues on the cytoplasmic domain of the receptor. The phosphorylated tyrosine residues can bind to two Src homology domains, and STAT molecules containing these domains are then recruited to the receptor. The phosphorylated and activated STAT molecules then form heterodimers or homodimers, which translocate to the nucleus and begin the transcriptional activation of target genes [11]. The predominant role of JAK/STAT signaling has been successively discovered in the development of inflammatory infiltration, immune regulation, and fibrosis [12].

## 2. Molecular Structure of JAK and STAT

**Figure 1:**
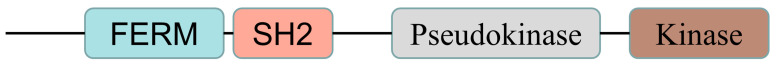
The structure of JAK. Schematic illustrating the four functional domains of JAK, FERM, SH2, pseudokinase, and Kinase. Figure prepared using information from various sources [20,21].

JAKs are non-receptor tyrosine kinases and consist of TYK2, JAK3, JAK2, and JAK1 in mammals [13]. JAK2, JAK1, and TYK2 are widely expressed in various tissues and cells, whereas JAK3 is expressed in the bone marrow and lymphoid systems [14]. Functionally, JAKs are composed of four domains: FERM, SH2, Pseudokinase, and Kinase (Figure 1) [15]. The FERM domain is mainly responsible for the binding of JAK kinases to the intracellular Box 1 portion of cytokine receptors. The SH2 domain is associated with the binding of FERM to the cytokine receptors, providing the binding site for the intracellular Box 2 of the cytokine receptors [16,17,18]. The pseudokinase domain has a structure similar to the kinase domain but does not have the biological function of the kinase domain. While no current consensus has been reached on pseudokinase domain research, more and more data have revealed that the pseudokinase domain has regulatory functions on the kinase domain [19]. The last and most important domain is the kinase domain, which is responsible for the phosphorylation of cytokines and downstream STAT molecules.

**Figure 2:**
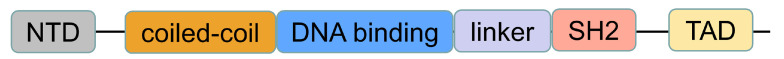
The structure of STAT. Schematic illustrating the conserved domain structure of STAT, including N-terminal domain (NTD), coiled-coil domain, DNA binding domain, transcriptional activation domain, SH2 domain, tyrosine activation domain (TAD). Figure prepared using information from various sources [20,26].

There are seven members of the STAT family in mammals, including STAT1, STAT2, STAT3, STAT4, STAT5a, STAT5b, and STAT6 [22,23]. Structurally, six conserved domains make up STATs: transcriptional activation domain, SH2 domain, DNA binding domain, coiled-coil domain, tyrosine activation domain (TAD), and amino-terminal domain (Figure 2) [24]. The amino-terminal domain is structurally independent and can bind to the GAS element together with the DNA-binding domain to induce homodimerization and nuclear translocation of unactivated STATs. The coiled-coil domain is rich in hydrophilic surfaces that can bind to regulatory factors. The DNA-binding domain is the central region controlling the selective DNA binding of each STAT molecule. The SH2 domain (also present in JAK molecules) is located at the dimer interface, is highly conserved among STAT molecules, and plays a key role in STAT signaling by recruiting STATs to the activated receptor complex. The TAD is adjacent to the SH2 domain and prevents autophosphorylation. The transcriptional activation domain is located at the carboxy terminus and varies widely among STAT members, determining various transcriptional regulations of STATs. The tyrosine-705 residue of STATs is an important site for their activation, and all STAT members except STAT2 and STAT6 have another phosphorylation site, serine-727 [25]. Normally, after activation by tyrosine phosphorylation, most STAT molecules can form homodimers and translocate to the nucleus, where they function as transcription factors.

## 3. Activation of the JAK/STAT Signaling Pathway

**Figure 3:**
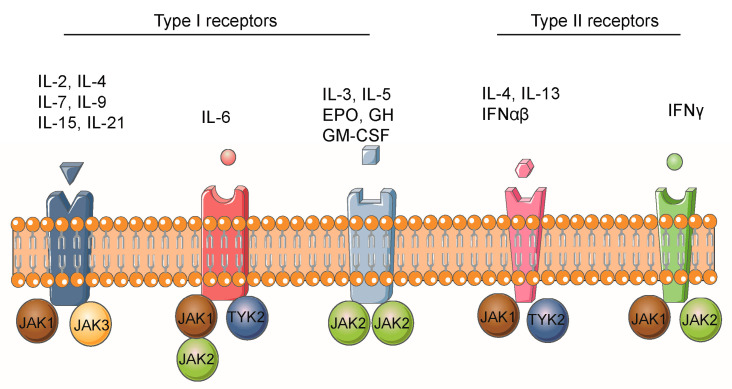
The activation of the JAK/STAT signaling pathway by cytokines. Schematic illustrating the preference of cytokines to bind JAK family. Figure prepared using information from various sources [21,34].

The binding of cytokines to their corresponding transmembrane receptors is the main trigger of the JAK/STAT signaling pathway, consequently launching intracellular signal transduction events and leading to altered gene expression. A variety of cytokines and growth factors can activate the JAK/STAT signaling pathway, including the interferon (IFN) family (including IFN-α, IFN-β, IFN-γ, IFN-κ, IFN-ω, IFN-ε, IFN-λ), the interleukin (IL)-10 family (including IL-10, IL-19, IL-20, IL-22, IL-24, IL-26), the gp130 family (including IL-11, IL-6, IL-12, IL-23, and granulocyte colony-stimulating factor), the γC family (including IL-2, IL-3, IL-4, IL-5, IL-7, IL-9, IL-15, and IL-21), and single-chain families (including thrombopoietin, prolactin, growth hormone(GH), and erythropoietin) [10,11,27,28,29]. The cytokine receptors that activate the JAK/STAT signaling pathway are mainly classified into type I and type II. Type I cytokine receptors bind and respond to cytokines through four α-helical strands and share a common amino acid motif (WSXWS). Type II cytokine receptors are similar to type I but lack the WSXWS motif. JAK1 is essential for signaling through these type II receptor complexes [30]. Typically, receptors required for hematopoietic cell development and proliferation prefer to bind to JAK2. The common γ-chain receptors prefer to bind to JAK1 and JAK3, whereas other receptors bind only to JAK1 [11]. Different receptor classes preferentially bind to one or multiple JAK family members (Figure 3). For example, IFN-α or IFN-β and their receptors stimulate STAT1, STAT2, STAT3, and STAT5 via JAK1 and TYK2, whereas IFN-γ stimulates STAT1, STAT3, and STAT5 via JAK1 and JAK2. All of the gp130 family except IL-12 and IL-23 stimulate STAT1, STAT3 and STAT5 via JAK1, JAK2 and TYK2, while IL-12 and IL-23 stimulate STAT3 and STAT4 via JAK2 and TYK2 [31,32]. After binding, the receptor undergoes a conformational change that brings JAK close to the proximal binding site of the receptor, and then the tyrosine residues on the cytoplasmic domain of the receptor are phosphorylated by the JAK molecule, recruiting STATs with the SH2 domain capable of binding these phosphotyrosine residues to the receptor. The activated STATs are then phosphorylated and dimerized, and the dimers translocate to the nucleus and activate the transcription of corresponding target genes. In addition to JAK-dependent activation via tyrosine, STATs can also be activated by pathways that are not JAK-dependent, such as via epidermal growth factor (EGF), platelet-derived growth factor (PDGF), extracellular signal-regulated kinase (ERK), protein kinase C, and mitogen-activated protein kinase (MAPK) [33]. Some growth factors also appear to be associated with specific STATs, for instance, colony-stimulating factor 1 and EGF stimulate STAT1, STAT3, and STAT5, whereas PDGF stimulates STAT1 and STAT3, and GH and prolactin stimulate only STAT5 [10,27].

## 4. The Roles of JAK/STAT in Hepatic Fibrosis

Hepatic fibrosis is a pathophysiological process in which various etiological factors such as cholestasis, viral infection, autoimmunity, and drug effects induce necrosis, apoptosis, and chronic damage to hepatocytes and the continued activation of certain cells, leading to abnormal proliferation of connective tissues in the liver, which in the long run forms hepatic fibrosis [35]. The activation of the JAK/STAT signaling pathway in the liver is mainly triggered by GH, cytokines [36], growth factors [37], and viral proteins [38]. Following liver injury, various cell subpopulations localized in the liver (Kupffer cells, hepatic stellate cells, hepatocytes, natural killer cells, dendritic cells, and lymphocytes) produce a range of cytokines with inflammatory or hepatoprotective potential. Among these cytokines, IFN-γ, IFN-α/β, IL-6, and IL-22 can activate the JAK/STAT signaling pathway [36]. The summary for JAK/STAT in hepatic fibrosis was seen in Table 1.

### 4.1. STAT1

STAT1 is currently considered as a negative regulator in hepatic fibrosis. STAT1 knockout mice had significantly accelerated disease progression in the CCl4-induced hepatic fibrosis model, compared with the control [39]. The antifibrotic effect of STAT1 originates from inhibited proliferation, increased apoptosis, and blocked cell cycle of hepatic stellate cells [39]. STAT1 inhibition plays a key role in reversing activated hepatic stellate cells [40]. It was shown that the inhibited liver regeneration after polyinosinic-polycytidylic acid (poly I:C) treatment post partial hepatectomy was associated with STAT1 activation and increased expression of interferon regulatory factor 1 in the hepatocyte [41], and subsequent studies confirmed that STAT1 terminated the therapeutic effects of poly I:C, suggesting that STAT1 is a negative regulator of liver regeneration [42]. However, STAT1 in hepatocytes functions promotes liver inflammation, injury, and fibrosis in some studies [43,44,45,46]. IFN-γ primarily activates STAT1 signaling and plays a key role in the immune response. IFN-γ overexpressing transgenic mice developed chronic hepatitis. Hep3B hepatocytes overexpressing STAT1 were more susceptible to IFN-γ-induced cell death [43,47,48]. STAT1 activation in hepatocytes is thought to be a pro-apoptotic signal, which increases cell death and promotes liver injury [49], while STAT3 activation inhibits the pro-inflammatory signaling of STAT1 and protects the liver from damage. Interestingly, activated STAT1 and STAT3 have opposite roles in liver pathophysiology (inflammation, injury, regeneration, and fibrosis), with STAT1 activation being detrimental to the liver and STAT3 activation being protective [49]. Indeed, STAT1 and STAT3 negatively regulate each other by inducing inhibitors of cytokine signaling 1 and cytokine signaling 3. In a model of concanavalin A-induced hepatitis, activated STAT1 exacerbates its induction of hepatitis [50], whereas activated STAT3 attenuated hepatitis [51]. Subsequent studies found that blocking the activation of hepatic STAT1 by genetic modification of certain genes prevented liver injury but blocking the activation of hepatic STAT3 exacerbated liver injury [52,53].

### 4.2. STAT2

Although STAT2 has an important role in antiviral immune responses [54,55,56], STAT2 can only be activated by the IFN family [57,58] and its role in hepatic injury and fibrosis has been studied very little. Ibrahim et al. conducted a study showing that STAT2 and IRF7 were significantly upregulated in patients with advanced hepatic fibrosis compared to those with early hepatic fibrosis [59]. In another study characterizing molecular changes associated with early hepatic fibrosis, STAT2/IRF9 expression was found to be increased in diseased livers of patients with hepatic fibrosis, compared with healthy control livers [60]. Recently, IL-27 was also found to induce STAT1 and STAT2, thereby inducing the expression of interferon-regulated proteins such as IRF-1, IRF-9, myxovirus resistance A, and guanylate-binding protein 2 to promote antiviral responses in hepatocytes and hepatic cancer cells [61]. All the aforementioned studies suggest a protective role of the STAT2 transcription factor in the progression of hepatic fibrosis.

### 4.3. STAT3

The role of STAT3 in hepatic fibrosis is controversial, as conflicting findings on STAT3 in hepatic fibrosis exist. In the CCl4-induced chronic model, silencing hepatocyte STAT3 significantly increased liver injury and inflammation [62], whereas in the acute CCl4 administration model, silencing STAT3 in hepatocytes reduced liver inflammation [63]. Liver tissue sections from patients with cirrhosis and liver cancer showed significantly elevated levels of both forms of STAT3 (phosphorylated and non-phosphorylated) [64]. JAK2/STAT3 signaling was significantly upregulated in the diethylnitrosamine-induced hepatic fibrosis of rats. Several studies have also found that JAK1/STAT3 interacts with the SMAD pathway to exacerbate hepatic fibrosis through TGF-β [65,66]. S-allyl-cysteine (one of the major antioxidants in aged garlic extracts) attenuates CCl4-induced hepatic fibrosis in rats, with mechanisms of action related to reduced SMAD3 and phosphorylation of STAT3 [67]. Primary hepatic stellate cells were stimulated with IL-6, and the increased protein of α-SMA and type I collagen α-1correlated with increased STAT3 phosphorylation, whereas STAT3 inhibitor (S3I-201) was capable to reverse the fibrotic phenotype of hepatic stellate cells [68]. In addition, the amelioration of CCl4-induced hepatic fibrosis by cucurbitacin-B is also associated with the blockade of STAT3 phosphorylation [69]. Whereas other findings support a protective effect of STAT3 on hepatic fibrosis. IL-22 protects against hepatic fibrosis by activating STAT3 in hepatocytes to facilitate cell survival and proliferation, and in hepatic stellate cells to promote cell senescence [70,71]. Metalloproteinase-1 levels were significantly reduced in hepatocyte-specific STAT3 knockout mice with CCl4-induced hepatic fibrosis [72]. It was suggested that metalloproteinase-1 is a downstream target of STAT3, and it is against both acute and chronic liver injuries [73]. The reason for these contradictory results may be the heterogeneity of studies on various hepatic fibrosis models.

### 4.4. STAT4

The roles of STAT4 in hepatic fibrosis are also bidirectional. Numerous findings suggest a protective role of IL-12/STAT4 in hepatic fibrosis, with key roles in tissue inflammation, fibrogenesis, and viral defense [74,75]. Phosphorylation of STAT4 is dependent on IFN-γ. Therefore, reduced IFN-γ secretion leads to impaired STAT4 phosphorylation, which in turn can result in liver inflammation and fibrosis in patients [76]. Genetic variants of STAT4 may also contribute to an increased risk of host fibrosis. Genotyping of single nucleotide polymorphisms of STAT4 in 160 liver transplantation patients with recurrent hepatitis showed that advanced fibrosis progression was highly correlated with the STAT4-T allele [77]. In the hepatic fibrosis of Schistosoma infection, the anti-IL-12 treated group had larger granulomas and increased fibrosis [78]. Whereas other studies have shown a pro-fibrotic effect of IL-12/STAT4. For example, overexpression of IL-12 in hepatocytes causes liver injury [58], and IL-12 administration induce inflammation of the liver [79,80]. Taken together, IL-12/STAT4 activation in immune cells is a double-edged sword that may promote liver injury and fibrosis by causing inflammation in the liver but may also prevent infection and thus reduce liver injury and fibrosis.

### 4.5. STAT5

The STAT5a protein is encoded by a gene located on human chromosome 17, while STAT5b is encoded by a gene located on mouse chromosome 11. GH and interleukins (IL-2, IL-3, IL-5), as well as several other cytokines, can activate STAT5 [81]. STAT5 was shown to have hepatoprotective and antifibrotic effects in the mouse model of cholestasis [58]. Loss of STAT5 in hepatocytes enhanced GH-induced STAT3 activity and increased TGF-β levels after CCl4 intervention, and STAT5 deficiency also increased the sensitivity of Kupffer or hepatic stellate cells to TGF-β and pro-hepatic fibrosis [82]. Deficiency of STAT5 in hepatocytes causes activation of STAT1 and reduces liver regeneration after partial hepatectomy [83]. This evidence suggested that STAT5 could join with other dysregulated STAT family members to play a role in fibrosis.

### 4.6. STAT6

STAT6 has pro- and anti-inflammatory effects, and its role in hepatic fibrosis is controversial. Kaplan et al. found reduced collagen deposition and smaller granulomas in the liver of Schistosoma mansoni-infected STAT6-deficient mice, compared with wild-type mice [84]. IL-4 induced STAT6 activation and served as a pathogenic role in various models of liver injury. Significant upregulation of IL-4 was detected in the fibrotic livers of Schistosoma mansoni-infected baboons, while blockade of IL-4 resulted in significantly less hepatic fibrosis in mice infected with Schistosoma mansoni [85]. Studies pointed out that IL-4 and IL-13 is capable of protecting against ischemia/reperfusion and inhibits inflammation; subsequently, it prevents hepatic fibrosis in drug-induced liver injuries through STAT6 activation [49].

## 5. The Role of JAK/STAT in Renal Fibrosis

Renal fibrosis is a pathophysiologic process in the progression of multiple chronic kidney diseases to end-stage renal disease (uremia). The pathophysiological changes in renal fibrosis include macrophage infiltration and activation and subsequent production of multiple growth factors and cytokines that stimulate downstream cellular changes, including renal mesangial cell activation, fibroblast proliferation, extracellular matrix activation, and progressive apoptosis [86,87]. Severe interstitial inflammatory infiltration can occur early in renal obstruction, and Kuratsune et al. showed that STAT3 is activated in a rat model of unilateral ureteral obstruction [88]. Pang et al. subsequently showed that treatment with a specific STAT3 inhibitor, S3I-201, reduced pro-fibrotic markers in obstructive nephropathy [89].

Ischemia-reperfusion injury can also promote the transdifferentiation of renal epithelial cells to mesenchymal cells, promoting renal interstitial fibrosis and leading to chronic fibrosis in the kidney [90,91]. Yang et al. observed that phosphorylation of JAK2, STAT1 and STAT3 in rat kidneys was significantly elevated during renal ischemia and reperfusion injury, and treatment with AG490, a JAK2-selective inhibitor, immediately before and after renal ischemia and reperfusion significantly inhibited the expression of p-JAK2, p-STAT1, and p-STAT3. Pre-treatment with AG490 improved renal function and attenuated renal tubular epithelial cell apoptosis and necrosis, as well as macrophage infiltration into the interstitial tissue. In contrast, delaying AG490 treatment until 3 h after renal ischemia and reperfusion failed to improve renal function, suggesting that JAK/STAT signaling activation plays a role early in the process of renal ischemia and reperfusion injury [92]. Arany et al. also demonstrated that severe oxidative stress can lead to STAT3 phosphorylation in mice with renal ischemia and reperfusion injury through activation of the EGF receptor and JAK2 kinase [93]. On the other hand, inhibition of JAK2 or STAT3 can lead to the activation of ERKs and promote cell survival during severe oxidative stress [93,94]. Moreover, Yokota et al. found that STAT6 plays a major protective role during renal ischemia and reperfusion injury [95]. STAT6-/- mice exhibited a significant deterioration in renal function during renal ischemia and reperfusion injury, compared with wild-type mice, and cytokine staining of T cells obtained from STAT6-/- mice showed increased IFN-γ production and decreased IL-4 production compared with wild-type mice [95]. STAT4-/- mice showed a slight improvement in renal function in ischemic kidney injury, and their T cells exhibited reduced IFN-γ production and increased IL-4 production. These studies suggest that STAT6 has a protective effect on renal function after ischemic and reperfusion injury and that IL-4 deficiency may be the main mechanism for the significant deterioration of renal function in STAT6-/- mice after ischemic injury [95]. Although activation of the JAK/STAT signaling pathway in renal ischemia and reperfusion injury has been demonstrated, the exact mechanism of its action remains unclear, and further studies are needed.

Additionally, high blood glucose levels stimulate many pro-inflammatory and pro-fibrotic factors, and diabetic nephropathy can lead to further development of glomerulosclerosis and tubulointerstitial fibrosis as proteinuria worsens [96,97]. The JAK/STAT signaling pathway is implicated in the pathophysiology of diabetic nephropathy and has been extensively studied in models of diabetic nephropathy [98]. Berthier et al. used a transcriptomic approach to describe the JAK/STAT signaling pathway in kidney tissue specimens from patients with early and progressive diabetic nephropathy [99]. In the renal tubular interstitial region, microarray analysis identified several JAK/STAT family members with downregulated expression in patients with early diabetic nephropathy, whereas most JAK/STAT family members such as JAK1, JAK2, JAK3, STAT1, STAT3, STAT4, and STAT5B were upregulated in patients with progressive diabetic nephropathy compared with controls. In contrast, in the glomeruli of the kidney, most JAK/STAT family members were upregulated in patients with early diabetic nephropathy and downregulated in patients with progressive diabetic nephropathy. Compared with the microarray results, messenger RNA (mRNA) expression of JAK1, JAK2, JAK3, STAT1, and STAT3 was increased in glomeruli from patients with early and progressive diabetic nephropathy, whereas mRNA expression was increased in the tubulointerstitial compartment only in patients with progressive diabetic nephropathy. Finally, the glomerular filtration rate in patients with early and progressive diabetic nephropathy was negatively correlated with JAK1, JAK2, JAK3, and STAT1 mRNA expression in the tubulointerstitial compartment, while there was no correlation between glomerular filtration rate and JAK/STAT activation in the glomeruli of the kidney [99]. These findings suggest that enhancement of the JAK/STAT signaling pathway may play an important role in diabetic nephropathy and is negatively correlated with renal function. In a rat model of streptozotocin-induced diabetes mellitus, Banes et al. demonstrated that high glucose levels induced the activation of JAK2, STAT1, STAT3, and STAT5 through an angiotensin-dependent mechanism [100], and in addition, AG490 treatment reduced urinary protein excretion in these animals. Similarly, Lu et al. explored the role of STAT3 in streptozotocin-induced diabetes in mice with reduced STAT3 activity [101]. Mice with 25% STAT3 activity had significantly reduced proteinuria, mesangial expansion, glomerular cell proliferation, and macrophage infiltration compared with mice with 75% STAT3 activity. Compared with mice with 75% STAT3 activity, the mRNA expression of IL-6, monocyte chemotactic protein-1, nuclear factor κB, type IV collagen, TGF-β1, and intercellular adhesion molecule 1, as well as the protein level of TGF-β1 and the level of type IV collagen fibers, were also significantly reduced in mice with 25% STAT3 activity [101]. The aforementioned results suggest that STAT3 has a key role in the progression of inflammatory cell infiltration, interstitial fibrosis, and abnormal matrix synthesis during early diabetic nephropathy. In an in vitro model, high glucose levels also enhanced angiotensin II-induced phosphorylation of STAT5B, STAT5A, STAT3, STAT1, and JAK2 in glomerular mesangial cells. Angiotensin II-induced glomerular mesangial cell growth (hyperplasia and hypertrophy) in a high-glucose environment stimulated type IV collagen synthesis and TGF-β1 and fibronectin production [102,103]. Treatment with AG490 reduced JAK2, STAT1, and STAT3 tyrosine phosphorylation and blocked glucose-induced TGF-β1 and fibronectin production in glomerular mesangial cells [103].

**Figure 4:**
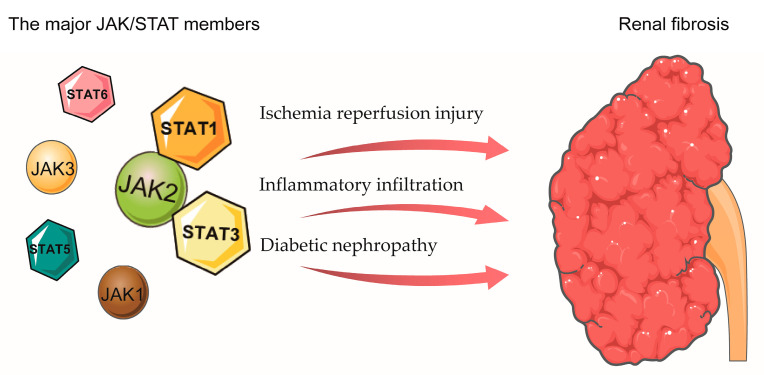
The role of JAK/STAT in renal fibrosis. Schematic illustrating the main JAK/STAT members may involve in renal fibrosis, primarily through ischemia reperfusion injury, inflammatory infiltration, and diabetic nephropathy to modulate renal fibrosis.

Moreover, the levels of p-STAT3 were increased in proliferating mesangial cells in a rat model of anti-Thy 1.1 glomerulonephritis (mesangial proliferative glomerulonephritis) [104,105]. In a mouse model of lupus nephritis manifested by immune complex deposition, glomerular cell proliferation, and inflammatory infiltration, p-JAK2 and p-STAT1 expression were significantly increased compared with normal control mice, with p-STAT1 expressed only in renal tubular epithelial cells, mesangial cells, and glomerular cells. AG490 treatment significantly reduced STAT1 and JAK2 activity in mice and decreased the expression of class II major histocompatibility complexes, IFN-γ, and monocyte chemotactic protein-1; it also reduced proteinuria and improved renal function [106]. These results suggest that the JAK/STAT signaling pathway plays an important role in renal interstitial fibrosis, extracellular matrix protein deposition, and further exacerbation of renal inflammation [107] (Figure 4).

## 6. The Role of JAK/STAT in Cardiac Fibrosis

The JAK/STAT signaling pathway has also been implicated in cardiac remodeling and cellular inflammatory responses [108]. JAK1, JAK2, STAT1, and STAT3 were activated in a murine model of cardiac ischemic preconditioning [109,110,111]. Cardiac hypertrophy and fibrosis are associated with the activation of the JAK2/STAT3 signaling pathway. Rats with the cardiac-specific knockout of STAT3 also exhibited ventricular remodeling and heart failure [112]. Dai et al. reported that high glucose-induced STAT3 and STAT1 phosphorylation in cardiac fibroblasts thereby leads to cardiac fibroblast proliferation and collagen fiber I and III synthesis [113]. ERK1/2 also acts in conjunction with STAT3 and STAT1 in cardiac fibroblasts to regulate collagen fibril synthesis and cell proliferation [113]. It has also been recently shown that PDGF/JAK-STAT signal transduction has an important role in atrial fibrotic remodeling and that PDGF stimulates upregulation of JAK-STAT expression and activity in atrial fibroblasts and enhances extracellular matrix protein production. Both the JAK2 selective inhibitor AG 1296 and STAT3 inhibitor S3I-201 attenuate the pro-fibroblastic effects of PDGF [114]. In addition, acute pressure overload and mechanical stress can activate JAK1, JAK2, TYK2, STAT2, STAT3, and the IL-6 family (including cardiotrophin 1, LIF, and IL-6 itself), and it has been suggested that this activation is a potentially important mechanism of myocardial hypertrophy [115]. The Cytokines of the IL-6 family can inhibit apoptosis and induce compensatory cellular hypertrophy via gp130 and STAT3, which in turn leads to dilated cardiomyopathy and heart failure [116,117]. In addition, the JAK/STAT pathway mediated cardioprotection through inducible nitric oxide synthase and cyclooxygenase 2 [109,118]. Thus, the JAK/STAT signaling pathway is also closely linked to cardiac fibrosis.

## 7. The Role of JAK/STAT in Myelofibrosis

Myelofibrosis is a BCR-ABL1-negative myeloproliferative neoplasm. In 2005, it was found that myelofibrosis was associated with the V617 mutation locus of JAK2 [119]. The JAK2 V617F mutation disrupts the self-repressive nature of the JH2 pseudokinase domain, leading to sustained activation of JAK2 kinase and STAT-mediated transcription. Subsequently, with a better understanding of this disease, it was found that most patients have upregulation of the JAK/STAT pathway due to at least one of the three genes JAK2, MPL, or CALR [120]. In vivo studies have found that injection of thrombopoietin induces megakaryocytosis, myelofibrosis, splenomegaly, and anemia [121,122]. The binding of MPL to thrombopoietin leads to the activation of various tyrosine kinase pathways, especially JAK/STAT, as it is the main pathogenesis mechanism [123]. Moreover, the JAK2 V617 mutation has been found to be present in approximately 96% of patients with polycythemia vera, 50% of patients with primary thrombocytosis, and 60% of patients with primary myelofibrosis [124]. Considering the crucial role of JAK2 in the pathogenesis of myeloproliferative illness, JAK2 inhibitors, ruxolitinib, and fedratinib were approved as new treatments for myelofibrosis and polycythemia vera.

## 8. The Role of JAK/STAT in Pulmonary Fibrosis

Pulmonary fibrosis, the end-stage of interstitial lung disease, is characterized pathologically by the destruction of the lung parenchyma and massive extracellular matrix deposition. Idiopathic pulmonary fibrosis is one of the most common subtypes of interstitial lung disease. Hypotheses about the pathogenesis of IPF are evolving while it remains complex and unclear. Initially, the hypothesis suggested that IPF is a disease with a dysregulated wound-healing response [125,126]. This hypothesis suggested that IPF is an excessive, uncontrolled wound healing response that is caused by fibrosis and whose main initial features are the massive infiltration of inflammatory cells and the release of pro-fibrotic products. This hypothesis advocates the use of glucocorticoids and azathioprine as first-line treatments for IPF. Unfortunately, the application of these two drugs has been proven to suppress immune functions without improving patient symptoms, even to the point of worsening the patient’s prognosis. A randomized, controlled, double-blind clinical trial showed that the combination of N-acetylcysteine, prednisone, and azathioprine was associated with higher mortality and hospitalization rates in IPF compared to patients in the placebo group over the same period of time [127]. The latest theory suggests that IPF is a process of recurrent lung injury and ineffective injury repair. Recent studies suggest that IPF is primarily triggered by alveolar epithelial cell injury, which causes apoptosis and leads to a series of downstream events that result in tissue fibrosis and remodeling [128,129].

Several descriptions of aberrant activity of JAK/STAT have been reported. Milara et al. evaluated 12 IPF patients and 10 corresponding healthy lung tissues by PCR quantification, protein immunoblotting, and immunohistochemistry and found that JAK2, p-JAK2, STAT3, and p-STAT3 were upregulated in lung tissues from IPF patients [130]. JAK2 is also elevated in patients with IPF-induced pulmonary arterial hypertension [130]. Others have analyzed lung tissues from seven patients with systemic sclerosis with interstitial lung disease and found that p-JAK1, p-JAK2, p-JAK3, and p-STAT3 were significantly increased compared to healthy controls [131,132]. Studies revealed that JAK/STAT was associated with anti-fibrotic effects. For example, dexamethasone attenuates the bleomycin-induced pulmonary fibrosis model via TGF-β, SMAD3, and JAK/STAT pathways, indirectly suggesting that the JAK/STAT pathway is associated with pulmonary fibrosis [133]. Similarly, systemic treatment with resveratrol significantly improved the lung condition in rats with adjuvant arthritis and interstitial lung disease through the JAK/STAT/RANKL pathway [134]. JAK1/STAT3 interactions with TGF-β have recently been shown to modulate myofibroblast transdifferentiation and fibrosis [135]. The JAK inhibitors presented a promising effect of reducing extracellular matrix deposition and fibroblast differentiation in animal models. The dual inhibitor of JAK2 and STAT3, JSI-124, attenuated bleomycin-induced pulmonary fibrosis in vivo [130]. The use of either the JAK2 inhibitor JSI-124 or small RNA interference of JAK2 inhibited the transition of pulmonary artery endothelial cells to fibroblasts in vitro, and in vivo models also confirmed that JAI-124 attenuated pulmonary fibrosis-induced pulmonary artery remodeling [136]. Moreover, others have recently used a JAK3 inhibitor to treat a model of pulmonary fibrosis and found a significant reduction in collagen deposition and fibrosis in lung tissues [137]. The Phase II clinical trial of the non-selective JAK inhibitor for IPF is undergoing. JAK/STAT is widely expressed in cells and regulated primary cellular biological processes. However, phenotypic studies have failed to answer how JAK/STAT in different cell types regulate pulmonary fibrosis now. Therefore, exploring the specific role of JAK/STAT in the fibrotic lungs will be required in the future.

## 9. Conclusions

JAK/STAT is closely associated with fibrosis in a variety of tissues and organs and has been most extensively studied in liver fibrosis. Studies in myelofibrosis have been applied clinically, and JAK inhibitors have been clinically approved for the treatment of myelofibrosis. In contrast, studies in cardiac, renal, and pulmonary fibrosis are scarce. Further studies exploring the mechanistic role of JAK/STAT in fibrosis are necessary, given the wide availability of various pan-JAK inhibitors, selective inhibitors, and the few inhibitors that have been used for clinical treatment. The treatment of fibrotic diseases is still a pressing challenge; however, JAK/STAT is closely related to fibrotic diseases and could be a target for the future treatment of fibrotic diseases.

## Figures and Tables

**Table 1:** The roles of STAT family in hepatic fibrosis.

STAT Proteins	Major Triggers or Cytokines	Possible Roles in Fibrotic Liver
STAT1	IFN-α, IFN-β, IFN-γ	Hepatic Stellate Cells→Loss→Inhibited proliferation, increased apoptosis and blocked cell cycle Hepatocytes→Inhibition of liver regeneration, and pro-apoptosis
STAT2	IFN-α, IFN-β, IFN-λ	Promote antiviral responses Protect against hepatic fibrosis
STAT3	IL-6, IL-22	Hepatocytes→Loss→Increased inflammation in the CCl4-induced chronic model Hepatocytes→Loss→Reduced inflammation in the CCl4-induced acute model
STAT4	IL-12, IFN-γ	Impaired STAT4 phosphorylation→liver inflammation and fibrosis Promotes liver inflammation
STAT5	GH, IL-2, IL-3, IL-5	Hepatocytes→Loss→Increased TGF-β levels, increased the sensitivity of Kupffer or hepatic stellate cells to TGF-β Hepatocytes→Loss→Reduces proliferation Antifibrotic effects in the mouse model of cholestasis
STAT6	IL-4, IL-13	Hepatocytes→Loss→Reduced collagen deposition Hepatic Stellate Cells→Promotes liver fibrogenesis Promotes inflammation in hepatitis Protects against ischemia/reperfusion and inflammation of drug-induced liver injuries

References are cited in the text.

## Data Availability

Not applicable.

## References

[1] Henderson N.C., Rieder F., Wynn T.A. (2020). Fibrosis: From mechanisms to medicines. Nature.

[2] Lv W., Booz G.W., Wang Y., Fan F., Roman R.J. (2018). Inflammation and renal fibrosis: Recent developments on key signaling molecules as potential therapeutic targets. Eur. J. Pharmacol..

[3] Mohammed S., Thadathil N., Selvarani R., Nicklas E.H., Wang D., Miller B.F., Richardson A., Deepa S.S. (2021). Necroptosis contributes to chronic inflammation and fibrosis in aging liver. Aging Cell.

[4] Sivakumar P., Das A.M. (2008). Fibrosis, chronic inflammation and new pathways for drug discovery. Inflamm. Res..

[5] Eming S.A., Wynn T.A., Martin P. (2017). Inflammation and metabolism in tissue repair and regeneration. Science.

[6] Darnell J.E., Kerr I.M., Stark G.R. (1994). Jak-STAT pathways and transcriptional activation in response to IFNs and other extracellular signaling proteins. Science.

[7] Jatiani S.S., Baker S.J., Silverman L.R., Reddy E.P. (2010). Jak/STAT pathways in cytokine signaling and myeloproliferative disorders: Approaches for targeted therapies. Genes Cancer.

[8] Bromberg J., Darnell J.E. (2000). The role of STATs in transcriptional control and their impact on cellular function. Oncogene.

[9] Bromberg J.F., Wrzeszczynska M.H., Devgan G., Zhao Y., Pestell R.G., Albanese C., Darnell J.E. (1999). Stat3 as an oncogene. Cell.

[10] Kisseleva T., Bhattacharya S., Braunstein J., Schindler C.W. (2002). Signaling through the JAK/STAT pathway, recent advances and future challenges. Gene.

[11] Murray P.J. (2007). The JAK-STAT signaling pathway: Input and output integration. J. Immunol..

[12] Bharadwaj U., Kasembeli M.M., Robinson P., Tweardy D.J. (2020). Targeting Janus Kinases and Signal Transducer and Activator of Transcription 3 to Treat Inflammation, Fibrosis, and Cancer: Rationale, Progress, and Caution. Pharmacol. Rev..

[13] Schindler C., Darnell J.E. (1995). Transcriptional responses to polypeptide ligands: The JAK-STAT pathway. Annu. Rev. Biochem..

[14] Gnanasambandan K., Sayeski P.P. (2011). A structure-function perspective of Jak2 mutations and implications for alternate drug design strategies: The road not taken. Curr. Med. Chem..

[15] Bousoik E., Montazeri Aliabadi H. (2018). “Do We Know Jack” About JAK? A Closer Look at JAK/STAT Signaling Pathway. Front. Oncol..

[16] Manning G., Whyte D.B., Martinez R., Hunter T., Sudarsanam S. (2002). The protein kinase complement of the human genome. Science.

[17] LaFave L.M., Levine R.L. (2012). JAK2 the future: Therapeutic strategies for JAK-dependent malignancies. Trends Pharm. Sci..

[18] Schindler C., Strehlow I. (2000). Cytokines and STAT signaling. Adv. Pharm..

[19] Hammaren H.M., Ungureanu D., Grisouard J., Skoda R.C., Hubbard S.R., Silvennoinen O. (2015). ATP binding to the pseudokinase domain of JAK2 is critical for pathogenic activation. Proc. Natl. Acad. Sci. USA.

[20] Hu X., Li J., Fu M., Zhao X., Wang W. (2021). The JAK/STAT signaling pathway: From bench to clinic. Signal Transduct. Target. Ther..

[21] Morris R., Kershaw N.J., Babon J.J. (2018). The molecular details of cytokine signaling via the JAK/STAT pathway. Protein Sci. Publ. Protein Soc..

[22] Bohmer F.D., Friedrich K. (2014). Protein tyrosine phosphatases as wardens of STAT signaling. JAKSTAT.

[23] Swiatek-Machado K., Kaminska B. (2020). STAT Signaling in Glioma Cells. Adv. Exp. Med. Biol..

[24] Yu H., Jove R. (2004). The STATs of cancer-new molecular targets come of age. Nat. Rev. Cancer.

[25] Decker T., Kovarik P. (2000). Serine phosphorylation of STATs. Oncogene.

[26] Levy D.E., Darnell J.E. (2002). Stats: Transcriptional control and biological impact. Nat. Rev. Mol. Cell Biol..

[27] O’Sullivan L.A., Liongue C., Lewis R.S., Stephenson S.E., Ward A.C. (2007). Cytokine receptor signaling through the Jak-Stat-Socs pathway in disease. Mol. Immunol..

[28] Yoshimura A., Naka T., Kubo M. (2007). SOCS proteins, cytokine signalling and immune regulation. Nat. Rev. Immunol..

[29] Aaronson D.S., Horvath C.M. (2002). A road map for those who don’t know JAK-STAT. Science.

[30] Ferrao R., Wallweber H.J., Ho H., Tam C., Franke Y., Quinn J., Lupardus P.J. (2016). The Structural Basis for Class II Cytokine Receptor Recognition by JAK1. Structure.

[31] Ozaki K., Leonard W.J. (2002). Cytokine and cytokine receptor pleiotropy and redundancy. J. Biol. Chem..

[32] Kotenko S.V., Langer J.A. (2004). Full house: 12 receptors for 27 cytokines. Int. Immunopharmacol..

[33] Abroun S., Saki N., Ahmadvand M., Asghari F., Salari F., Rahim F. (2015). STATs: An Old Story, Yet Mesmerizing. Cell J..

[34] Clark J.D., Flanagan M.E., Telliez J.B. (2014). Discovery and development of Janus kinase (JAK) inhibitors for inflammatory diseases. J. Med. Chem..

[35] Younossi Z.M., Loomba R., Anstee Q.M., Rinella M.E., Bugianesi E., Marchesini G., Neuschwander-Tetri B.A., Serfaty L., Negro F., Caldwell S.H. (2018). Diagnostic modalities for nonalcoholic fatty liver disease, nonalcoholic steatohepatitis, and associated fibrosis. Hepatology.

[36] Gao B. (2005). Cytokines, STATs and liver disease. Cell. Mol. Immunol..

[37] Ruff-Jamison S., Chen K., Cohen S. (1993). Induction by EGF and interferon-gamma of tyrosine phosphorylated DNA binding proteins in mouse liver nuclei. Science.

[38] Machida K., Tsukamoto H., Liu J.C., Han Y.P., Govindarajan S., Lai M.M., Akira S., Ou J.H. (2010). c-Jun mediates hepatitis C virus hepatocarcinogenesis through signal transducer and activator of transcription 3 and nitric oxide-dependent impairment of oxidative DNA repair. Hepatology.

[39] Jeong W.I., Park O., Radaeva S., Gao B. (2006). STAT1 inhibits liver fibrosis in mice by inhibiting stellate cell proliferation and stimulating NK cell cytotoxicity. Hepatology.

[40] Zhang H., Chen F., Fan X., Lin C., Hao Y., Wei H., Lin W., Jiang Y., He F. (2017). Quantitative Proteomic analysis on Activated Hepatic Stellate Cells reversion Reveal STAT1 as a key regulator between Liver Fibrosis and recovery. Sci. Rep..

[41] Sun R., Gao B. (2004). Negative regulation of liver regeneration by innate immunity (natural killer cells/interferon-gamma). Gastroenterology.

[42] Sun R., Park O., Horiguchi N., Kulkarni S., Jeong W.I., Sun H.Y., Radaeva S., Gao B. (2006). STAT1 contributes to dsRNA inhibition of liver regeneration after partial hepatectomy in mice. Hepatology.

[43] Hong F., Jaruga B., Kim W.H., Radaeva S., El-Assal O.N., Tian Z., Nguyen V.A., Gao B. (2002). Opposing roles of STAT1 and STAT3 in T cell-mediated hepatitis: Regulation by SOCS. J. Clin. Investig..

[44] Jaruga B., Hong F., Sun R., Radaeva S., Gao B. (2003). Crucial role of IL-4/STAT6 in T cell-mediated hepatitis: Up-regulating eotaxins and IL-5 and recruiting leukocytes. J. Immunol..

[45] Siebler J., Wirtz S., Klein S., Protschka M., Blessing M., Galle P.R., Neurath M.F. (2003). A key pathogenic role for the STAT1/T-bet signaling pathway in T-cell-mediated liver inflammation. Hepatology.

[46] Wen J., Zhou Y., Wang J., Chen J., Yan W., Wu J., Yan J., Zhou K., Xiao Y., Wang Y. (2020). Retraction Note: Interactions between Th1 cells and Tregs affect regulation of hepatic fibrosis in biliary atresia through the IFN-gamma/STAT1 pathway. Cell Death Differ..

[47] Kim W.H., Hong F., Radaeva S., Jaruga B., Fan S., Gao B. (2003). STAT1 plays an essential role in LPS/D-galactosamine-induced liver apoptosis and injury. Am. J. Physiol. Gastrointest Liver Physiol..

[48] Car B.D., Eng V.M., Schnyder B., Ozmen L., Huang S., Gallay P., Heumann D., Aguet M., Ryffel B. (1994). Interferon gamma receptor deficient mice are resistant to endotoxic shock. J. Exp. Med..

[49] Gao B., Wang H., Lafdil F., Feng D. (2012). STAT proteins—Key regulators of anti-viral responses, inflammation, and tumorigenesis in the liver. J. Hepatol..

[50] Torisu T., Nakaya M., Watanabe S., Hashimoto M., Yoshida H., Chinen T., Yoshida R., Okamoto F., Hanada T., Torisu K. (2008). Suppressor of cytokine signaling 1 protects mice against concanavalin A-induced hepatitis by inhibiting apoptosis. Hepatology.

[51] Park O., Wang H., Weng H., Feigenbaum L., Li H., Yin S., Ki S.H., Yoo S.H., Dooley S., Wang F.S. (2011). In vivo consequences of liver-specific interleukin-22 expression in mice: Implications for human liver disease progression. Hepatology.

[52] Klein C., Wustefeld T., Assmus U., Roskams T., Rose-John S., Muller M., Manns M.P., Ernst M., Trautwein C. (2005). The IL-6-gp130-STAT3 pathway in hepatocytes triggers liver protection in T cell-mediated liver injury. J. Clin. Investig..

[53] Lafdil F., Wang H., Park O., Zhang W., Moritoki Y., Yin S., Fu X.Y., Gershwin M.E., Lian Z.X., Gao B. (2009). Myeloid STAT3 inhibits T cell-mediated hepatitis by regulating T helper 1 cytokine and interleukin-17 production. Gastroenterology.

[54] Heim M.H., Thimme R. (2014). Innate and adaptive immune responses in HCV infections. J. Hepatol..

[55] Blaszczyk K., Olejnik A., Nowicka H., Ozgyin L., Chen Y.L., Chmielewski S., Kostyrko K., Wesoly J., Balint B.L., Lee C.K. (2015). STAT2/IRF9 directs a prolonged ISGF3-like transcriptional response and antiviral activity in the absence of STAT1. Biochem. J..

[56] Shrivastava S., Meissner E.G., Funk E., Poonia S., Shokeen V., Thakur A., Poonia B., Sarin S.K., Trehanpati N., Kottilil S. (2016). Elevated hepatic lipid and interferon stimulated gene expression in HCV GT3 patients relative to non-alcoholic steatohepatitis. Hepatol. Int..

[57] Orlent H., Reynaert H., Bourgeois S., Dideberg V., Adler M., Colle I., De Maeght S., Laleman W., Michielsen P., Moreno C. (2011). IL28B polymorphism and the control of hepatitis C virus infection: Ready for clinical use?. Acta Gastroenterol. Belg..

[58] Kong X., Horiguchi N., Mori M., Gao B. (2012). Cytokines and STATs in Liver Fibrosis. Front. Physiol..

[59] Ibrahim M.K., Khedr A., Bader El Din N.G., Khairy A., El Awady M.K. (2017). Increased incidence of cytomegalovirus coinfection in HCV-infected patients with late liver fibrosis is associated with dysregulation of JAK-STAT pathway. Sci. Rep..

[60] Bieche I., Asselah T., Laurendeau I., Vidaud D., Degot C., Paradis V., Bedossa P., Valla D.C., Marcellin P., Vidaud M. (2005). Molecular profiling of early stage liver fibrosis in patients with chronic hepatitis C virus infection. Virology.

[61] Bender H., Wiesinger M.Y., Nordhoff C., Schoenherr C., Haan C., Ludwig S., Weiskirchen R., Kato N., Heinrich P.C., Haan S. (2009). Interleukin-27 displays interferon-gamma-like functions in human hepatoma cells and hepatocytes. Hepatology.

[62] Wang H., Lafdil F., Wang L., Park O., Yin S., Niu J., Miller A.M., Sun Z., Gao B. (2011). Hepatoprotective versus oncogenic functions of STAT3 in liver tumorigenesis. Am. J. Pathol..

[63] Horiguchi N., Lafdil F., Miller A.M., Park O., Wang H., Rajesh M., Mukhopadhyay P., Fu X.Y., Pacher P., Gao B. (2010). Dissociation between liver inflammation and hepatocellular damage induced by carbon tetrachloride in myeloid cell-specific signal transducer and activator of transcription 3 gene knockout mice. Hepatology.

[64] Ding Y.F., Wu Z.H., Wei Y.J., Shu L., Peng Y.R. (2017). Hepatic inflammation-fibrosis-cancer axis in the rat hepatocellular carcinoma induced by diethylnitrosamine. J. Cancer Res. Clin. Oncol..

[65] Ogata H., Chinen T., Yoshida T., Kinjyo I., Takaesu G., Shiraishi H., Iida M., Kobayashi T., Yoshimura A. (2006). Loss of SOCS3 in the liver promotes fibrosis by enhancing STAT3-mediated TGF-beta1 production. Oncogene.

[66] Tang L.Y., Heller M., Meng Z., Yu L.R., Tang Y., Zhou M., Zhang Y.E. (2017). Transforming Growth Factor-beta (TGF-beta) Directly Activates the JAK1-STAT3 Axis to Induce Hepatic Fibrosis in Coordination with the SMAD Pathway. J. Biol. Chem..

[67] Gong Z., Ye H., Huo Y., Wang L., Huang Y., Huang M., Yuan X. (2018). S-allyl-cysteine attenuates carbon tetrachloride-induced liver fibrosis in rats by targeting STAT3/SMAD3 pathway. Am. J. Transl. Res..

[68] Kagan P., Sultan M., Tachlytski I., Safran M., Ben-Ari Z. (2017). Both MAPK and STAT3 signal transduction pathways are necessary for IL-6-dependent hepatic stellate cells activation. PLoS ONE.

[69] Sallam A.M., Esmat A., Abdel-Naim A.B. (2018). Cucurbitacin-B attenuates CCl4 -induced hepatic fibrosis in mice through inhibition of STAT-3. Chem. Biol. Drug Des..

[70] Khawar M.B., Azam F., Sheikh N., Abdul Mujeeb K. (2016). How Does Interleukin-22 Mediate Liver Regeneration and Prevent Injury and Fibrosis?. J. Immunol. Res..

[71] Kong X., Feng D., Wang H., Hong F., Bertola A., Wang F.S., Gao B. (2012). Interleukin-22 induces hepatic stellate cell senescence and restricts liver fibrosis in mice. Hepatology.

[72] Wang H., Lafdil F., Wang L., Yin S., Feng D., Gao B. (2011). Tissue inhibitor of metalloproteinase 1 (TIMP-1) deficiency exacerbates carbon tetrachloride-induced liver injury and fibrosis in mice: Involvement of hepatocyte STAT3 in TIMP-1 production. Cell Biosci..

[73] Kasembeli M.M., Bharadwaj U., Robinson P., Tweardy D.J. (2018). Contribution of STAT3 to Inflammatory and Fibrotic Diseases and Prospects for its Targeting for Treatment. Int. J. Mol. Sci..

[74] Edlich B., Ahlenstiel G., Zabaleta Azpiroz A., Stoltzfus J., Noureddin M., Serti E., Feld J.J., Liang T.J., Rotman Y., Rehermann B. (2012). Early changes in interferon signaling define natural killer cell response and refractoriness to interferon-based therapy of hepatitis C patients. Hepatology.

[75] Wang Y., Qu A., Wang H. (2015). Signal transducer and activator of transcription 4 in liver diseases. Int. J. Biol. Sci..

[76] El Sharkawy R., Thabet K., Lampertico P., Petta S., Mangia A., Berg T., Metwally M., Bayoumi A., Boonstra A., Brouwer W.P. (2018). A STAT4 variant increases liver fibrosis risk in Caucasian patients with chronic hepatitis B. Aliment. Pharm..

[77] Jiang D.K., Ma X.P., Wu X., Peng L., Yin J., Dan Y., Huang H.X., Ding D.L., Zhang L.Y., Shi Z. (2015). Genetic variations in STAT4,C2,HLA-DRB1 and HLA-DQ associated with risk of hepatitis B virus-related liver cirrhosis. Sci. Rep..

[78] Cheng Y.L., Song W.J., Liu W.Q., Lei J.H., Kong Z., Li Y.L. (2012). The effects of interleukin (IL)-12 and IL-4 deficiency on worm development and granuloma formation in Schistosoma japonicum-infected mice. Parasitol. Res..

[79] Harada N., Shimada M., Okano S., Suehiro T., Soejima Y., Tomita Y., Maehara Y. (2004). IL-12 gene therapy is an effective therapeutic strategy for hepatocellular carcinoma in immunosuppressed mice. J. Immunol..

[80] Chang C.J., Chen Y.H., Huang K.W., Cheng H.W., Chan S.F., Tai K.F., Hwang L.H. (2007). Combined GM-CSF and IL-12 gene therapy synergistically suppresses the growth of orthotopic liver tumors. Hepatology.

[81] Mair M., Blaas L., Osterreicher C.H., Casanova E., Eferl R. (2011). JAK-STAT signaling in hepatic fibrosis. Front. Biosci..

[82] Hosui A., Kimura A., Yamaji D., Zhu B.M., Na R., Hennighausen L. (2009). Loss of STAT5 causes liver fibrosis and cancer development through increased TGF-{beta} and STAT3 activation. J. Exp. Med..

[83] Cui Y., Hosui A., Sun R., Shen K., Gavrilova O., Chen W., Cam M.C., Gao B., Robinson G.W., Hennighausen L. (2007). Loss of signal transducer and activator of transcription 5 leads to hepatosteatosis and impaired liver regeneration. Hepatology.

[84] Kaplan M.H., Schindler U., Smiley S.T., Grusby M.J. (1996). Stat6 is required for mediating responses to IL-4 and for development of Th2 cells. Immunity.

[85] Farah I.O., Mola P.W., Kariuki T.M., Nyindo M., Blanton R.E., King C.L. (2000). Repeated exposure induces periportal fibrosis in Schistosoma mansoni-infected baboons: Role of TGF-beta and IL-4. J. Immunol..

[86] Liu Y. (2004). Epithelial to mesenchymal transition in renal fibrogenesis: Pathologic significance, molecular mechanism, and therapeutic intervention. J. Am. Soc. Nephrol..

[87] Humphreys B.D. (2018). Mechanisms of Renal Fibrosis. Annu. Rev. Physiol..

[88] Kuratsune M., Masaki T., Hirai T., Kiribayashi K., Yokoyama Y., Arakawa T., Yorioka N., Kohno N. (2007). Signal transducer and activator of transcription 3 involvement in the development of renal interstitial fibrosis after unilateral ureteral obstruction. Nephrology.

[89] Pang M., Ma L., Gong R., Tolbert E., Mao H., Ponnusamy M., Chin Y.E., Yan H., Dworkin L.D., Zhuang S. (2010). A novel STAT3 inhibitor, S3I-201, attenuates renal interstitial fibroblast activation and interstitial fibrosis in obstructive nephropathy. Kidney Int..

[90] Menke J., Sollinger D., Schamberger B., Heemann U., Lutz J. (2014). The effect of ischemia/reperfusion on the kidney graft. Curr. Opin. Organ Transpl..

[91] Carvajal G., Rodriguez-Vita J., Rodrigues-Diez R., Sanchez-Lopez E., Ruperez M., Cartier C., Esteban V., Ortiz A., Egido J., Mezzano S.A. (2008). Angiotensin II activates the Smad pathway during epithelial mesenchymal transdifferentiation. Kidney Int..

[92] Yang N., Luo M., Li R., Huang Y., Zhang R., Wu Q., Wang F., Li Y., Yu X. (2008). Blockage of JAK/STAT signalling attenuates renal ischaemia-reperfusion injury in rat. Nephrol. Dial. Transpl..

[93] Arany I., Megyesi J.K., Nelkin B.D., Safirstein R.L. (2006). STAT3 attenuates EGFR-mediated ERK activation and cell survival during oxidant stress in mouse proximal tubular cells. Kidney Int..

[94] Neria F., Castilla M.A., Sanchez R.F., Gonzalez Pacheco F.R., Deudero J.J., Calabia O., Tejedor A., Manzarbeitia F., Ortiz A., Caramelo C. (2009). Inhibition of JAK2 protects renal endothelial and epithelial cells from oxidative stress and cyclosporin A toxicity. Kidney Int..

[95] Yokota N., Burne-Taney M., Racusen L., Rabb H. (2003). Contrasting roles for STAT4 and STAT6 signal transduction pathways in murine renal ischemia-reperfusion injury. Am. J. Physiol. Ren. Physiol..

[96] Hasslacher C. (1989). Diabetic nephropathy: Structural-functional relationships. Contrib. Nephrol..

[97] Schrijvers B.F., De Vriese A.S., Flyvbjerg A. (2004). From hyperglycemia to diabetic kidney disease: The role of metabolic, hemodynamic, intracellular factors and growth factors/cytokines. Endocr. Rev..

[98] Marrero M.B., Banes-Berceli A.K., Stern D.M., Eaton D.C. (2006). Role of the JAK/STAT signaling pathway in diabetic nephropathy. Am. J. Physiol. Ren. Physiol..

[99] Berthier C.C., Zhang H., Schin M., Henger A., Nelson R.G., Yee B., Boucherot A., Neusser M.A., Cohen C.D., Carter-Su C. (2009). Enhanced expression of Janus kinase-signal transducer and activator of transcription pathway members in human diabetic nephropathy. Diabetes.

[100] Banes A.K., Shaw S., Jenkins J., Redd H., Amiri F., Pollock D.M., Marrero M.B. (2004). Angiotensin II blockade prevents hyperglycemia-induced activation of JAK and STAT proteins in diabetic rat kidney glomeruli. Am. J. Physiol. Ren. Physiol..

[101] Lu T.C., Wang Z.H., Feng X., Chuang P.Y., Fang W., Shen Y., Levy D.E., Xiong H., Chen N., He J.C. (2009). Knockdown of Stat3 activity in vivo prevents diabetic glomerulopathy. Kidney Int..

[102] Amiri F., Shaw S., Wang X., Tang J., Waller J.L., Eaton D.C., Marrero M.B. (2002). Angiotensin II activation of the JAK/STAT pathway in mesangial cells is altered by high glucose. Kidney Int..

[103] Wang X., Shaw S., Amiri F., Eaton D.C., Marrero M.B. (2002). Inhibition of the Jak/STAT signaling pathway prevents the high glucose-induced increase in tgf-beta and fibronectin synthesis in mesangial cells. Diabetes.

[104] Hirai T., Masaki T., Kuratsune M., Yorioka N., Kohno N. (2006). PDGF receptor tyrosine kinase inhibitor suppresses mesangial cell proliferation involving STAT3 activation. Clin. Exp. Immunol..

[105] Yanagita M., Arai H., Nakano T., Ohashi K., Mizuno K., Fukatsu A., Doi T., Kita T. (2001). Gas6 induces mesangial cell proliferation via latent transcription factor STAT3. J. Biol. Chem..

[106] Wang S., Yang N., Zhang L., Huang B., Tan H., Liang Y., Li Y., Yu X. (2010). Jak/STAT signaling is involved in the inflammatory infiltration of the kidneys in MRL/lpr mice. Lupus.

[107] Matsui F., Meldrum K.K. (2012). The role of the Janus kinase family/signal transducer and activator of transcription signaling pathway in fibrotic renal disease. J. Surg. Res..

[108] Fan Z., Gao Y., Huang Z., Xue F., Wu S., Yang J., Zhu L., Fu L. (2018). Protective effect of hydrogen-rich saline on pressure overload-induced cardiac hypertrophyin rats: Possible role of JAK-STAT signaling. BMC Cardiovasc. Disord..

[109] Xuan Y.T., Guo Y., Han H., Zhu Y., Bolli R. (2001). An essential role of the JAK-STAT pathway in ischemic preconditioning. Proc. Natl. Acad. Sci. USA.

[110] Xuan Y.T., Guo Y., Zhu Y., Han H., Langenbach R., Dawn B., Bolli R. (2003). Mechanism of cyclooxygenase-2 upregulation in late preconditioning. J. Mol. Cell. Cardiol..

[111] Xuan Y.T., Guo Y., Zhu Y., Wang O.L., Rokosh G., Messing R.O., Bolli R. (2005). Role of the protein kinase C-epsilon-Raf-1-MEK-1/2-p44/42 MAPK signaling cascade in the activation of signal transducers and activators of transcription 1 and 3 and induction of cyclooxygenase-2 after ischemic preconditioning. Circulation.

[112] Hilfiker-Kleiner D., Hilfiker A., Fuchs M., Kaminski K., Schaefer A., Schieffer B., Hillmer A., Schmiedl A., Ding Z., Podewski E. (2004). Signal transducer and activator of transcription 3 is required for myocardial capillary growth, control of interstitial matrix deposition, and heart protection from ischemic injury. Circ. Res..

[113] Dai B., Cui M., Zhu M., Su W.L., Qiu M.C., Zhang H. (2013). STAT1/3 and ERK1/2 synergistically regulate cardiac fibrosis induced by high glucose. Cell Physiol. Biochem..

[114] Chen Y., Surinkaew S., Naud P., Qi X.Y., Gillis M.A., Shi Y.F., Tardif J.C., Dobrev D., Nattel S. (2017). JAK-STAT signalling and the atrial fibrillation promoting fibrotic substrate. Cardiovasc. Res..

[115] Pan J., Fukuda K., Saito M., Matsuzaki J., Kodama H., Sano M., Takahashi T., Kato T., Ogawa S. (1999). Mechanical stretch activates the JAK/STAT pathway in rat cardiomyocytes. Circ. Res..

[116] Hirota H., Yoshida K., Kishimoto T., Taga T. (1995). Continuous activation of gp130, a signal-transducing receptor component for interleukin 6-related cytokines, causes myocardial hypertrophy in mice. Proc. Natl. Acad. Sci. USA.

[117] Ancey C., Menet E., Corbi P., Fredj S., Garcia M., Rucker-Martin C., Bescond J., Morel F., Wijdenes J., Lecron J.C. (2003). Human cardiomyocyte hypertrophy induced in vitro by gp130 stimulation. Cardiovasc. Res..

[118] Barry S.P., Townsend P.A., Latchman D.S., Stephanou A. (2007). Role of the JAK-STAT pathway in myocardial injury. Trends Mol. Med..

[119] Tefferi A., Lasho T.L., Schwager S.M., Steensma D.P., Mesa R.A., Li C.Y., Wadleigh M., Gary Gilliland D. (2005). The JAK2(V617F) tyrosine kinase mutation in myelofibrosis with myeloid metaplasia: Lineage specificity and clinical correlates. Br. J. Haematol..

[120] Schieber M., Crispino J.D., Stein B. (2019). Myelofibrosis in 2019: Moving beyond JAK2 inhibition. Blood Cancer J..

[121] Ulich T.R., del Castillo J., Senaldi G., Kinstler O., Yin S., Kaufman S., Tarpley J., Choi E., Kirley T., Hunt P. (1996). Systemic hematologic effects of PEG-rHuMGDF-induced megakaryocyte hyperplasia in mice. Blood.

[122] Yanagida M., Ide Y., Imai A., Toriyama M., Aoki T., Harada K., Izumi H., Uzumaki H., Kusaka M., Tokiwa T. (1997). The role of transforming growth factor-beta in PEG-rHuMGDF-induced reversible myelofibrosis in rats. Br. J. Haematol..

[123] He X., Chen Z., Jiang Y., Qiu X., Zhao X. (2013). Different mutations of the human c-mpl gene indicate distinct haematopoietic diseases. J. Hematol. Oncol..

[124] Zahr A.A., Salama M.E., Carreau N., Tremblay D., Verstovsek S., Mesa R., Hoffman R., Mascarenhas J. (2016). Bone marrow fibrosis in myelofibrosis: Pathogenesis, prognosis and targeted strategies. Haematologica.

[125] Maher T.M., Wells A.U., Laurent G.J. (2007). Idiopathic pulmonary fibrosis: Multiple causes and multiple mechanisms?. Eur. Respir. J..

[126] Wilson M.S., Wynn T.A. (2009). Pulmonary fibrosis: Pathogenesis, etiology and regulation. Mucosal. Immunol..

[127] Raghu G., Anstrom K.J., King T.E., Lasky J.A., Martinez F.J., Idiopathic Pulmonary Fibrosis Clinical Research Network (2012). Prednisone, azathioprine, and N-acetylcysteine for pulmonary fibrosis. N. Engl. J. Med..

[128] Coward W.R., Saini G., Jenkins G. (2010). The pathogenesis of idiopathic pulmonary fibrosis. Adv. Respir. Dis..

[129] Wolters P.J., Collard H.R., Jones K.D. (2014). Pathogenesis of idiopathic pulmonary fibrosis. Annu. Rev. Pathol..

[130] Milara J., Hernandez G., Ballester B., Morell A., Roger I., Montero P., Escriva J., Lloris J.M., Molina-Molina M., Morcillo E. (2018). The JAK2 pathway is activated in idiopathic pulmonary fibrosis. Respir. Res..

[131] Talotta R. (2021). The rationale for targeting the JAK/STAT pathway in scleroderma-associated interstitial lung disease. Immunotherapy.

[132] Wang W., Bhattacharyya S., Marangoni R.G., Carns M., Dennis-Aren K., Yeldandi A., Wei J., Varga J. (2020). The JAK/STAT pathway is activated in systemic sclerosis and is effectively targeted by tofacitinib. J. Scleroderma Relat. Disord..

[133] Shi K., Jiang J., Ma T., Xie J., Duan L., Chen R., Song P., Yu Z., Liu C., Zhu Q. (2014). Dexamethasone attenuates bleomycin-induced lung fibrosis in mice through TGF-beta, Smad3 and JAK-STAT pathway. Int. J. Clin. Exp. Med..

[134] Yang G., Lyu L., Wang X., Bao L., Lyu B., Lin Z. (2019). Systemic treatment with resveratrol alleviates adjuvant arthritis-interstitial lung disease in rats via modulation of JAK/STAT/RANKL signaling pathway. Pulm Pharm. Ther..

[135] Wang F., Wang S., Zhang C., Tian X., Zhou Y., Xuan W., Matteson E.L., Luo F., Tschumperlin D., Vassallo R. (2022). Noncanonical JAK1/STAT3 interactions with TGF-β modulate myofibroblast transdifferentiation and fibrosis. Am. J. Physiol. Lung Cell. Mol. Physiol..

[136] Milara J., Ballester B., Morell A., Ortiz J.L., Escriva J., Fernandez E., Perez-Vizcaino F., Cogolludo A., Pastor E., Artigues E. (2018). JAK2 mediates lung fibrosis, pulmonary vascular remodelling and hypertension in idiopathic pulmonary fibrosis: An experimental study. Thorax.

[137] Zhu Y., Zheng X., Wang C., Sun X., Sun H., Ma T., Li Y., Liu K., Chen L., Ma X. (2020). Synthesis and biological activity of thieno[3,2-d]yrimidines as potent JAK3 inhibitors for the treatment of idiopathic pulmonary fibrosis. Bioorganic Med. Chem..

